# Insights Into Eye Care Accessibility: Geospatial Distribution of Eye Care Providers and Socioeconomic Factors by ZIP Code

**DOI:** 10.1167/tvst.13.3.21

**Published:** 2024-03-26

**Authors:** Meagan T. Tran, Valeria V. Gonzalez, Carolyn Mead-Harvey, Joanne F. Shen

**Affiliations:** 1 Mayo Clinic Alix School of Medicine, Scottsdale, AZ, USA; 2 Mayo Clinic Department of Quantitative Health Sciences, Scottsdale, AZ, USA; 3 Mayo Clinic Department of Ophthalmology, Scottsdale, AZ, USA

**Keywords:** eye care access, social determinants, geographic distribution, epidemiology

## Abstract

**Purpose:**

In the United States, the ZIP Code has long been used to collect geospatial data revealing disparities in social determinants of health. This cross-sectional study examines the distribution of eye care access in association with local socioeconomic factors at a ZIP Code level.

**Methods:**

Data from the 2020 Centers of Medicare and Medicaid Services and American Community Survey were used to examine locations of 47,949 providers (17,631 ophthalmologists and 30,318 optometrists) and corresponding local socioeconomic variables (education, employment, and income). Multivariable zero-inflated negative binomial regression was used to model eye care provider count per capita in each ZIP Code area with socioeconomic factors as independent covariates.

**Results:**

For every 1% increase in percentage of population over 25 years with a bachelor's degree or higher, the expected number of providers increases by 4.4% (incidence rate ratio [IRR] = 1.044; 95% confidence interval [CI], 1.041–1.046; *P* < 0.001). For every 1% increase in percentage unemployment, the expected number of providers decreases by 2.7% (IRR = 0.973; 95% CI, 0.964–0.983; *P* < 0.001). However, for every $1000 increase in median household income, the expected number of providers decreases by 1.6% (IRR = 0.984; 95% CI, 0.983–0.986; *P* < 0.001).

**Conclusions:**

Disparities in access exist in areas of lower employment and educational attainment, as both have positive correlations with eye care provider access. Conversely, areas of greater median household income have lower access to providers.

**Translational Relevance:**

This research contributes to a greater field studying social determinants of health and may inform public health strategies on allocation of providers to improve equitable access to vision care.

## Introduction

The economic burden of vision loss in the United States is estimated to be $134.2 billion, with nursing home care, medical services, and reduced labor force composing the majority of the cost. Vision loss for a patient can equate to an average cost of $16,638 per year.^1^ However, many eye conditions are preventable if diagnosed and treated in a timely manner by an eye care provider (ECP), defined as an ophthalmologist or optometrist.^2^ Previous research has demonstrated this relationship between local ECP access and the visual health outcomes of a community.^3^^,^^4^ However, ECPs are currently distributed unequally across the United States,^5^^,^^6^ and some studies suggest that the disparity has increased over recent years.^7^ Although telemedicine has been suggested to expand access to visual screening, the technology does not yet exist for true eye exams and procedures to be conducted remotely, which calls into question patients’ ability to commute to more established eye care centers.^8^ Communities of disadvantaged socioeconomic status (SES) have long been shown to have poorer health outcomes, partly due to barriers in access to health care.^9^^,^^10^ Recent studies have shown an association between visual difficulty and social determinants of health (SDOH) such as educational attainment, insurance status, and food insecurity.^11^ Smaller scale studies have specifically demonstrated associations between SES factors and the prevalence of certain pathologies such as childhood strabismus,^12^ glaucoma,^13^^–^^16^ cataracts,^17^ and macular degeneration.^18^

In the United States, ZIP Codes and their equivalent geocodes have long been used to collect data in relation to geographic communities providing more precise and granular demographic data to understand local trends. Research has shown disparities spanning across economic, environmental, educational, and health factors related to geographic location. These disparities indicate that where a patient resides can thus be one of the most important factors contributing to their overall health outcomes. This has been shown in various fields of medicine such as diabetes,^19^^,^^20^ obesity rates,^21^ cancer,^22^ and maternal mortality.^23^

Few recent studies have shown geographic distribution among ophthalmology access, and those that exist examined larger scale geographies such as county-level data. Wang et al.^24^ studied the availability of eye care in California on a county level in relation to visual impairment. Additionally, Walsh et al.^7^ revealed disparities in access to pediatric ophthalmologists at the county level. However, in studying these geographic disparities in access, one must also consider the unique socioeconomic factors impacting the local patient populations. Thus, smaller geographic areas such as ZIP Codes allow for more precise measurements in comparing neighborhood characteristics.

Government policymakers are reforming health care to improve its administration, finances, and delivery. The U.S. Department of Health and Human Services’ first strategic goal is to protect and strengthen equitable access to high-quality and affordable health care.^25^ Current initiatives and strategies being implemented to address disparities in access to eye care include community-based programs such as mobile eye clinics^26^ and teleophthalmology.^27^ Although these platforms can offer convenience, increase access to basic eye care, and aid in triage and initial assessments, they may not fully replace comprehensive in-person eye care, especially for complex cases or advanced treatments.

The results of this study may inform the national strategy on the allocation and distribution of future public health intervention efforts to improve equity in vision health and access to eye care. There are state programs that provide incentives, such as loan replacements or scholarships, for optometrists to provide eye care for rural or underserved populations. Comparatively, there are more programs providing mental health services and dental services through incentive programs.^28^ We thus conducted a cross-sectional study to examine the association between the location of ECPs and local SES factors with the hypothesis that ZIP Code areas consisting of lower SES factors also have lower access to ECPs.

## Methods

Ophthalmologists and optometrists listed in the publicly available 2020 Centers for Medicare and Medicaid Services (CMS) were included, as well as their corresponding five-digit ZIP Codes.^29^ ZIP Codes were converted to ZIP Code tabulation areas (ZCTAs) using a 2020 ZIP to ZCTA crosswalk to match U.S. Census data to geographic area. ZCTAs with zero total population or of the type “post office or large volume customer” were excluded from analysis to remove areas that likely did not represent residential areas. Public data from the American Community Survey in 2020 published by the U.S. Census Bureau were used for population counts and selected social and economic characteristics, including the following:

1. Educational attainment—Percent of population 25 years and over with a bachelor's degree or higher
2. Employment status—Civilian labor force unemployment rate
3. Income level—Median household income in 2020 dollars.

Provider density outcomes were measured in units of number of providers per 100,000. This study was exempt from Institutional Review Board approval. Multivariable zero-inflated negative binomial regression was used to model ECP count per ZCTA with the following SES factors as covariates: percent racial or ethnic minority, educational attainment, employment status, and income level. Parameter estimates from the multivariable model are exponentiated to give the incidence rate ratio (IRR) for each variable. The IRRs were used to estimate relative counts of ECPs per unit increase in SES factors with a 95% confidence interval (CI). *P* < 0.001 was used as the threshold for statistical significance. Statistical analysis was performed using SAS 9.4. Data visualization was performed using Tableau Public software to map the geospatial distribution of ECPs.

## Results

A total of 47,949 ECPs were identified from the CMS database, which included 17,631 ophthalmologists and 30,318 optometrists. Summary statistics for the included ZCTAs are shown in Table 1. We initially reviewed 41,107 ZCTAs, of which 10,409 were excluded due to ZCTA type of “post office or large volume customer” or zero population count; 30,698 ZCTAs were included in the analysis. A sensitivity analysis included all ZCTAs with non-zero population (*n* = 32,830). Table 2 describes 14 ZCTAs excluded from analysis due to zero population count.

**Table 1.** Summary Statistics by ZCTA

	ZCTA (*N* = 30,698)
Population count, total	
Mean ± SD	10,981.2 ± 15,303.50
Median (IQR)	3607.0 (1019.0–15,673.0)
Range	1.0–126,310.0
Providers per 100,000	
Mean ± SD	10.9 ± 152.57
Median (IQR)	0.0 (0.0–6.2)
Range	0.0–15,436.2
Ophthalmology providers per 100,000	
Mean ± SD	4.7 ± 134.79
Median (IQR)	0.0 (0.0–0.0)
Range	0.0–15,436.2
Optometry providers per 100,000	
Mean ± SD	6.2 ± 51.53
Median (IQR)	0.0 (0.0–4.2)
Range	0.0–6250.0
Percent of population 25 years and over with bachelor's degree or higher^a^	
Mean ± SD	25.9 ± 16.90
Median (IQR)	21.4 (14.3–33.4)
Range	0.0–100.0
Civilian labor force unemployment rate^b^	
Mean ± SD	5.3 ± 5.52
Median (IQR)	4.3 (2.4–6.7)
Range	0.0–100.0
Household income (2020 dollars)^c^	
Mean ± SD	63,750.30 ± 26,624.63
Median (IQR)	58,229.00 (46,769.00–74,391.00)
Range	7428.00–248,091.00

a For this variable, 139 ZCTAs were missing information.

b For this variable, 99 ZCTAs were missing information.

c For this variable, 1352 ZCTAs were missing information.

**Table 2.** ECP ZCTAs With Zero Population

ZCTA	City, State	ECPs (Ophthalmology, Optometry), *n*	Associated Medical Center (If Applicable)
90095	Los Angeles, CA	87 (76, 11)	UCLA Stein Eye Institute
55905	Rochester, MN	56 (48, 8)	Mayo Clinic
75390	Dallas, TX	51 (45, 6)	UT Southwestern
33101	Miami, FL	34 (26, 8)	Bascom Palmer Eye Institute
17822	Danville, PA	19 (9, 10)	Geisinger Medical Center
53792	Madison, WI	10 (5, 5)	University of Wisconsin
85723	Tucson, AZ	3 (1, 2)	University of Arizona
96859	Honolulu, HI	3 (1, 2)	Tripler Army Medical Center
10020	New York, NY	2 (0, 2)	—
10170	New York, NY	2 (0, 2)	—
10174	New York, NY	2 (0, 2)	—
38132	Memphis, TN	2 (0, 2)	—
70836	Baton Rouge, LA	2 (0, 2)	—
90090	Los Angeles, CA	2 (0, 2)	—

**Figure 1.**
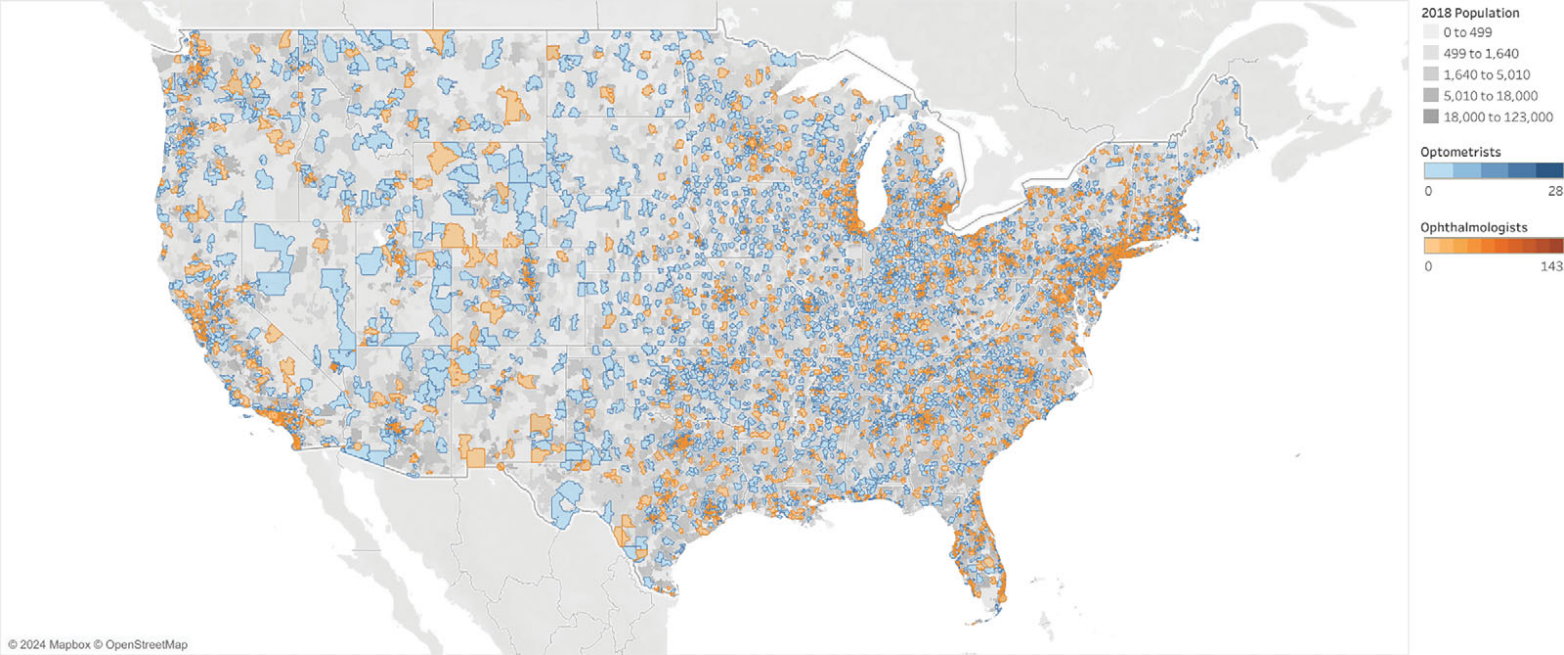
Map of optometrists (*blue*) and ophthalmologists (*orange*) in the contiguous United States by ZIP Code. Background map shading indicates 2018 population.

Most of these locations were large medical centers without a residential population, including Stein Eye Institute in Los Angeles, CA (87 ECPs); Mayo Clinic in Rochester, MN (56 ECPs); University of Texas Southwestern in Dallas, TX (51 ECPs); and Bascom Palmer Eye Institute in Miami, FL (34 ECPs). A map of ECPs in the United States, separated by profession, is shown in Figure 1 with a full interactive figure available online at the following link: https://public.tableau.com/views/EyeCareProvidersintheUnitedStates/Overlay?:language=en-US&:display_count=n&:origin=viz_share_link.

The pattern of distribution of providers generally follows population densities with higher concentrations around large metropolitan cities and coastal regions. In particular, Western states with more sparse population densities such as Nevada and South Dakota have greater areas without any ECP coverage. Table 3 shows the top 10 ZCTAs with the greatest number of combined providers. Note that the 90095 ZCTA had zero population and was thus excluded from further analysis. Although there is significant overlap between practicing optometrists and ophthalmologists, more rural areas have greater access to optometry care compared to ophthalmology.

**Table 3.** Top 10 ZIP Code Areas With the Greatest Number of ECPs

ZCTA	City, State	ECPs (Ophthalmology, Optometry), *n*	Population, *n*
02114	Boston, MA	171 (143, 28)	13,260
33136	Miami, FL	94 (81, 13)	17,065
10021	New York, NY	88 (83, 5)	42,253
90095	Los Angeles, CA	87 (76, 11)	0
21231	Baltimore, MD	85 (74, 11)	15,906
77030	Houston, TX	84 (79, 5)	11,362
48105	Ann Arbor, MI	79 (72, 7)	36,918
19107	Philadelphia, PA	68 (63, 5)	14,689
10032	New York, NY	66 (59, 7)	59,527
98104	Seattle, WA	63 (52, 11)	15,155

**Figure 2.**
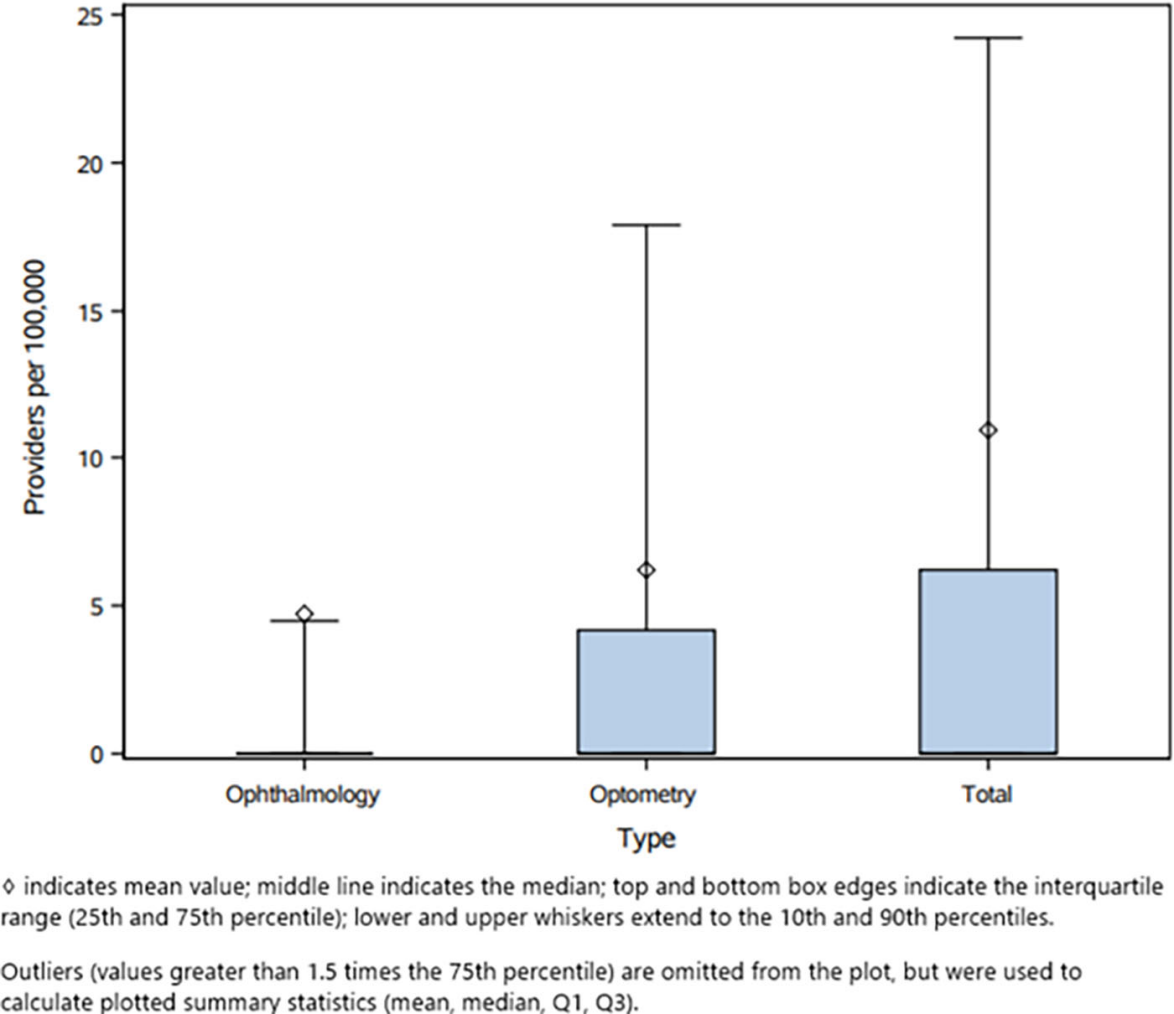
Box plot of provider rate per 100,000 by provider type.

Figure 2 visualizes the distribution of provider rates per 100,000. Provider rate per capita was highly variable across ZCTAs. A large percentage of ZCTAs (21,560/30,698, 70.2%) had zero providers. The median number of optometrists, ophthalmologists, and overall providers was 0. The 90th percentile for number of overall providers was 24.2 (4.5 for ophthalmology, 17.9 for optometry).

After adjusting for other SES factors using multivariable modeling, higher educational attainment (defined as percent of population 25 years or older holding a bachelor's degree or higher) was associated with greater access to local ECPs. For a 1% increase in percentage of population over 25 years with a bachelor's degree or higher, the expected number of ECPs increases by 4.4% (IRR = 1.044; 95% CI, 1.041–1.046; *P* < 0.001). When separating by type of provider, this trend remained consistent for both ophthalmologists (IRR = 1.059; 95% CI, 1.055–1.063; *P* < 0.001) and optometrists (IRR = 1.029; 95% CI, 1.026–1.031; *P* < 0.001).

After adjusting for other SES covariates, higher unemployment rates were associated with poorer local access to providers overall and to optometrists alone. For each 1% increase in percentage unemployment, the expected number of total ECPs decreases by 2.7% (IRR = 0.973; 95% CI, 0.964–0.983; *P* < 0.001). This association was also observed for optometry providers (IRR = 0.964; 95% CI, 0.954–0.974; *P* < 0.001), but the association was not statistically significant for ophthalmology providers (IRR = 0.994; 95% CI, 0.977–1.012; *P* = 0.50).

Finally, after adjusting for SES covariates, increasing median household income was associated with decreased access to ECPs. For an increase in median household income of $1000, the expected number of ECPs decreases by 1.6% (IRR = 0.984; 95% CI, 0.983–0.986; *P* < 0.001). This association was greater for ophthalmology providers (IRR = 0.9809; 95% CI, 0.978–0.982; *P* < 0.001) than optometry providers (IRR = 0.989; 95% CI, 0.988–0.990; *P* < 0.001).

Results from a sensitivity analysis that included all ZCTAs with population greater than zero, regardless of type, were similar to those described above for the analysis set that excluded ZCTAs from post offices or large volume customers.

## Discussion

### Status of ECP Distribution

The broad distribution of ECPs follows a bicoastal distribution with clusters around areas of high population densities. Optometrists are typically more evenly distributed across both urban and rural areas, whereas ophthalmologists are more likely to concentrate in metropolitan areas where there is greater access to advanced medical facilities and hospitals. Factors that may impact where ECPs choose to practice may include local quality education, cost-of-living, job opportunities, and the presence of academic centers. Further studies are needed to assess the influence of each factor on the decision by ECPs to practice in certain locations to then create public policy to increase the workforce in areas of need.

### Factors Influencing Distribution

Higher unemployment and lower educational attainment are associated with lower expected ECP availability, whereas there is unexpectedly greater ECP availability in areas with lower median household income. The distribution of ECPs can be influenced by a combination of factors such as population density, market demand, health care infrastructure, availability of resources, and historical patterns of health care delivery. Areas with lower median household income often have a higher proportion of individuals eligible for Medicaid and other safety net programs. Additionally, market forces may influence the distribution of health care providers such as potentially increased patient volume, lower overhead costs in lower income areas, or lower competition in areas of lower income. The usage of land may also differ between areas with lower median household income and higher median household income. Land from areas of higher median household income may be allocated for larger residential properties, leaving less availability for ECPs. In addition, ECP data for areas of higher median household income may not be available if they are only accepting private insurances. These factors can be complex and may not be directly correlated with the income levels of an area.

Further studies are needed to examine regional differences in urban versus rural areas. Although there may be a higher concentration of ECPs in areas of lower median household income, disparities in access to eye care services can still exist. Affordability, insurance coverage, transportation, and other barriers can limit the ability of individuals in these areas to access the eye care they need, despite the presence of providers. Addressing these barriers comprehensively is crucial to ensuring equitable access to eye care services for all populations.

### Limitations

Provider data were available only for providers who accepted patients on Medicare and Medicaid in 2020. CMS data primarily captures information on health care professionals who participate in Medicare and Medicaid programs and may not include those who do not participate in these programs or who treat patients exclusively outside of these programs. This includes those who focus on private-pay patients or work in non-traditional settings. The number of ophthalmologists in this study (17,631) accounted for over 90% of total actively practicing ophthalmologists (18,948) in the United States as reported by the Association of American Medical Colleges in 2022.^30^ The number of optometrists in this study (30,318), however, represented only 82% of the estimated total number of optometrists reported by the U.S. Bureau of Labor Statistics in 2020 (36,690).^31^ Therefore, there is a greater reliability in ophthalmology statistics than those for optometry, as a lesser percentage of optometry providers bill through Medicare or Medicaid.

Information on specific practice patterns (e.g., private practice vs. academic centers) was not available for analysis in this study. Few studies exist examining ophthalmologic practice patterns and their relevance to public health, but a study during the COVID-19 pandemic showed that private practices offered quicker availability for cataract evaluations than university centers.^32^ Moreover, the growing number of private-equity acquisitions of ophthalmology private practices may further complicate the type of care available for patients of varying SES; however, the majority of acquisitions occur in metropolitan areas, affecting a greater proportion of private insurance coverage.^33^^,^^34^ In the American Academy of Ophthalmology's most recent survey of its membership, over 70% of respondents reported working in a private practice, with 98% accepting Medicare patients.^35^ Further studies will be necessary to examine the relationship between practice patterns and patient care and SDOH characteristics.

When conducting a cross-sectional census study and utilizing ZIP Codes or ZCTAs for geospatial analysis, it is essential to consider their inherent limitations.^36^^,^^37^ ZIP Codes are primarily designed for mail delivery purposes and may not accurately reflect the true geographic boundaries of communities or neighborhoods. This can lead to potential misclassification or oversimplification of the study area, potentially obscuring important spatial variations within ZIP Code boundaries; however, ZIP Codes can still serve as a useful tool when linked to census data. Despite not aligning perfectly with true community boundaries, ZIP Codes provide a standardized geographic unit that facilitates data aggregation and comparison across different areas. They offer a practical and readily available method for researchers to access relevant demographic and socioeconomic data from the census. Moreover, although ZIP Codes may not capture all of the complexities within a specific area, they can still provide valuable insights into broad spatial patterns and help identify potential disparities or trends. By acknowledging the limitations and combining ZIP Code data with other complementary information, we can gain a more nuanced understanding of the study area and its associated characteristics.

### Policy and Planning Considerations

This research contributes to a greater field studying community SDOH and may inform national public health strategy on the allocation and distribution of providers to improve access to eye care. Understanding the geographic distribution of ECPs in relation to SDOH can inform health care planning, resource allocation, and workforce development strategies. Identifying areas with limited access can help target interventions and policies to improve the availability and affordability of eye care for underserved populations. Our current data suggest that areas of low educational attainment are associated with having the least access to both ophthalmologists and optometrists. Expanding access to these areas must also consider the patient population’s health literacy.

In this study, it is evident that the number of optometrists in the CMS database is small compared to the total number of practicing optometrists reported by the Bureau of Labor Statistics. However, despite this limitation, the findings shed light on the current state of access to eye care for patients who rely on Medicare and Medicaid. The significance of these results becomes even more pronounced when considering the ongoing debates surrounding the scope of practice in optometry.^38^ It is crucial to comprehend which patient populations will be impacted by potential changes in scope, as this knowledge can play a vital role in shaping policy decisions at both the federal and state levels. By understanding the specific demographics affected by scope changes, policymakers can make informed decisions to ensure equitable and accessible eye care services for all individuals, regardless of their insurance coverage.

Information on the status of eye care access can guide policymakers and health care administrators in determining where to allocate resources such as eye care clinics, vision screening programs, or mobile eye care units to address disparities and improve access for underserved communities. Initiatives can be designed to provide education and awareness programs in areas with low educational attainment, employment assistance for low-income individuals to improve affordability of eye care, or community-based programs that address specific social determinants influencing eye health outcomes. Moreover, understanding the difference in distribution between optometrists and ophthalmologists can allow for collaboration and coordination among health care providers to enhance overall eye care delivery.

### Future Directions

We chose to study local education attainment, employment, and median household income based on the availability of data; however, we also recognize that other social determinants not captured by this study often interact with and influence each other. These include access to transportation, social support networks, quality housing, exposure to pollution, internet access, and insurance coverage. Future research directions must further investigate the distribution of ECPs considering additional variables or incorporating longitudinal data.

Overall, researching the geographic location of ECPs in relation to local social determinants of health provides valuable insights into disparities in access to eye care services and helps inform strategies to reduce these disparities, improve eye health outcomes, and promote health equity. Ongoing monitoring and evaluation of ECP distribution can inform targeted interventions and policy adjustments.
